# Epigenetic information loss is a common feature of multiple diseases and aging

**DOI:** 10.1007/s11357-025-01767-7

**Published:** 2025-07-11

**Authors:** Naor Sagy, Chieh Chang, Maayan Gal, Daniel Z. Bar

**Affiliations:** Department of Oral Biology, Goldschleger School of Dental Medicine, Gray Faculty of Medical and Health Sciences, Tel Aviv University, 69978 Tel Aviv, Israel

**Keywords:** Aging, Epigenetic information loss, Pathological conditions, DNA methylation

## Abstract

Aging is a major risk factor for a plethora of diseases. The information theory of aging posits that epigenetic information loss is a principal driver of the aging process. Despite this, the connection between epigenetic information loss and disease has not been thoroughly investigated. Here, we analyzed tissue-unique methylation patterns in healthy and diseased human organs, revealing that for several diseases these patterns degrade, regressing to a mean form. We interpret this as epigenetic information loss, where tissue-unique patterns erode. Information loss is not limited to diseases. Age-related erosion of unique methylation patterns was observed in some tissues and cells, while other tissues and cells diverged away from the mean. Our findings demonstrate that analyzing methylation patterns in tissue-unique sites can effectively distinguish between patients and healthy controls at least in some diseases, and underscore the role of epigenetic information loss as a common feature in various pathological conditions.

**Graphical abstract:**

Tissue unique methylation pattern regress toward the mean upon disease. A single methylation site, showing low methylation in the liver and high in every other tissue, becomes more methylated in diseased livers.

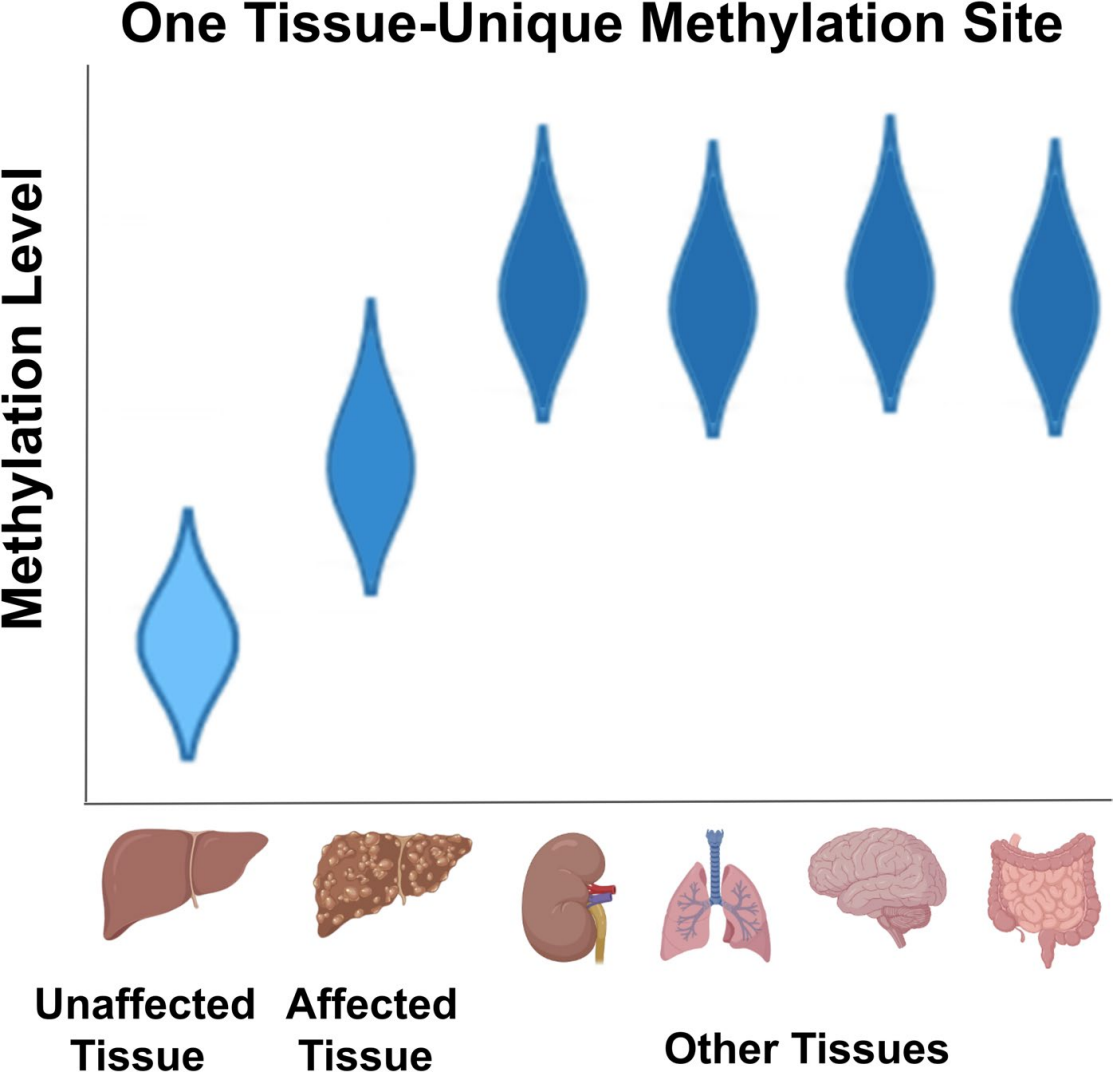

**Supplementary Information:**

The online version contains supplementary material available at 10.1007/s11357-025-01767-7.

## Introduction

Aging is the primary risk factor for numerous chronic diseases, such as cardiovascular diseases, cancer, and type 2 diabetes (T2D) [1]. Epigenetic alterations, including changes in methylation levels at specific genomic loci, are a well-known hallmark of aging [2–4]. Linear combinations of these loci, known as epigenetic clocks, strongly correlate with chronological age [5] and can predict all-cause mortality [6]. Behaviors that correlate with decreased healthspan and lifespan, including smoking and bad diet, accelerate some epigenetic clocks [6]. Stress may also induce rapid increase in biological age, as measured by epigenetic clocks [7]. Interestingly, recovery from stress may revert these changes [7]. Similarly, heterochronic parabiosis reduced the epigenetic age [8]. However, the causality of these methylation changes and why certain loci correlate with age remain unresolved [2].

The epigenetic theory of aging posits that the loss of epigenetic information disrupts youthful gene expression patterns, leading to cellular dysfunction and senescence [9–12]. Thus, information loss is suggested to drive the aging process [13]. Age-dependent loss of youthful gene expression patterns has been demonstrated in some cases [14–16], but not in others [17]. By contrast, single-cell level analysis of global coordination also suggests that transcriptional dysregulation is common or universal with aging [18].

Medical diagnosis typically relies on prior knowledge and the identification of relevant pathologies to explain symptoms. Specific DNA methylation patterns strongly correlate with medical conditions, but these correlations are only inferred by comparing methylation patterns of labeled cohorts [19–22]. In this study, we demonstrate that tissue-specific methylation patterns change with age and the progression of certain diseases. This pattern change allows us to predict chronic kidney disease (CKD) and sun exposure in healthy skin using methylation data alone, without any additional information or prior knowledge. Moreover, in multiple cases, the direction of change is not random; instead, it moves towards a common form across all other tissues, which we interpret as epigenetic information loss. Our findings establish the loss of epigenetic information as a shared feature of multiple diseases.

## Results

### Identification of tissue-specific methylation sites

Epigenetic information loss can take many forms, and can be challenging to identify and quantify in a consistent manner across multiple diseases, tissues and datasets. It was previously shown that in the kidney, methylation sites with a kidney-unique signature always regress towards the common form with CKD progression [23]. The unique methylation signature of the kidney, as manifested in these sites, erodes as the tissue becomes more similar to the rest of the body. The existence of tissue-unique methylation sites allows the exploration of such information loss, in a manner that is both tissue-specific and consistent across all examined tissues.

**Fig. 1:**
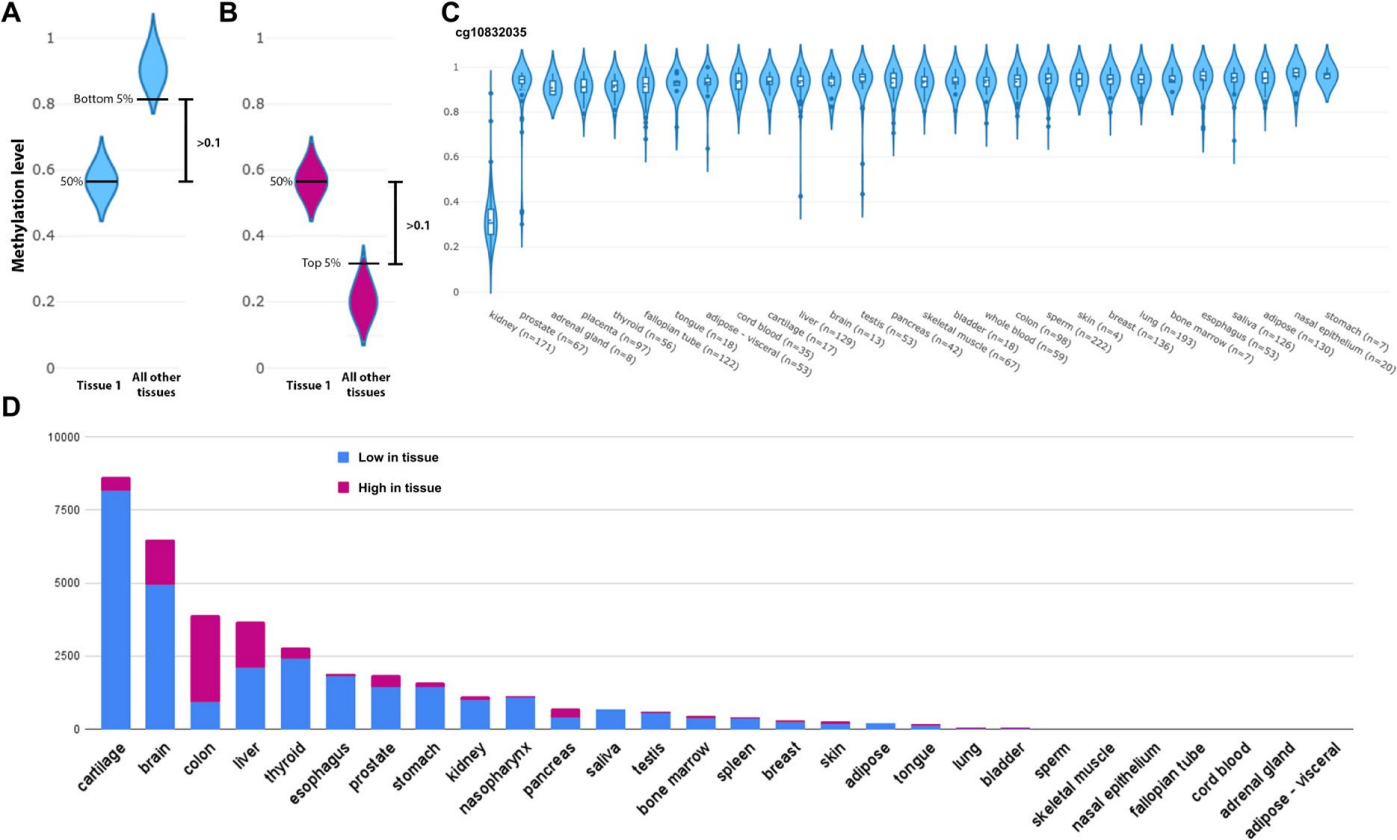
Identification of tissue-specific methylation sites. Methylation sites that show a median absolute methylation value at least 0.1 below the bottom 5% quantile (**A**) or above the top 5% quantile (**B**) are considered unique. For example (**C**) shows a unique methylation pattern in the kidney. (**D**) Unique methylation sites for each of the tissues, excluding the placenta, that had over 40,000 low and 10,000 high sites

To facilitate this analysis, we generated a dataset of all human tissue-specific methylation sites (Fig. 1A–C) based on the human NGDC-CNCB dataset [24]. Of the 30 tissues across 5323 samples in the dataset, 23 had unique methylation sites, ranging from 55 in bladder to 50,872 in placenta (Fig. 1D; Supp. Table 1). In total, 87,922, or ~ 18% of sites tested, displayed a unique methylation pattern in at least one tissue, with over half of them in the placenta. We note that while conceptually similar to other reported tissue-specific and cell-type specific methylation patterns [25], due to technical differences including selection criteria, directionality and dataset used, the dataset significantly differs in the eventual sites selected. Thus, we also use a different terminology to avoid confusion.

### Hypothesis-free mapping of kidney-unique methylation sites correlates with CKD

**Fig. 2:**
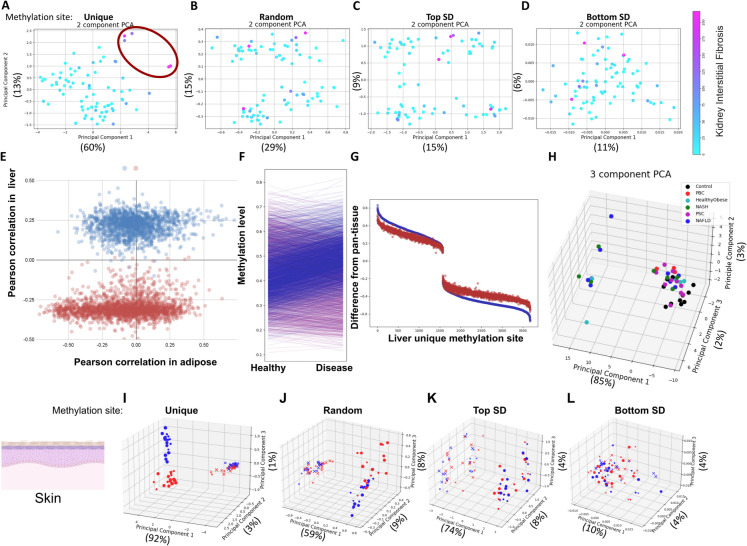
Epigenetic information loss in disease and stress. PCA analysis of kidney-unique methylation sites clusters kidneys with high levels of interstitial fibrosis (**A**), but not when random (**B**), high variability (**C**) or low variability (**D**) sites are used. (**E**) Liver-unique methylation sites show a binomial distribution of correlations to liver diseases (healthy = 0; disease = 1) in liver samples (Y axis), but not in adipose (X axis). More-methylated samples (red) show a negative correlation, while less-methylated samples (blue) show a positive correlation. (**F**) Methylation levels (mean) for sites that are more (red) and less (blue) methylated in the liver (compared to the rest of the body) reveals regression to the mean in disease. (**G**) Differences between each liver-unique methylation site mean and pan-tissue average, in healthy (blue) and sick (red) samples. Regression to the mean (red closer to zero) is observed in nearly all sites. (**H**) PCA of liver-unique methylation sites in men only. Separation based on tissue-unique sites following environmental stress**:** 3D PCA analysis of skin-unique methylation sites separated sun-exposed (red) from sun-protected (blue) samples (**I**), but not when random (**J**), high variability (**K**) or low variability (**L**) sites are used. X—dermis; o—epidermis; size indicates young (small) or old (large). In each plot, the number of sites in the control groups was selected to match the unique group

Epigenetic information loss occurs in nearly all uniquely methylated sites as CKD progresses ([23] and Supp. Tables 2–3). To obtain an unbiased, global view of these data, we projected the methylation values of kidney sites (Supp. Tables 1–3) into a 2D space using principal component analysis (PCA). This mapping spatially separated individuals with high and low interstitial fibrosis (Fig. 2A). To determine if this is a general feature across all methylation sites, we repeated the analysis with randomly selected methylation sites (Fig. 2B) and extended the controls to sites with high (Fig. 2C) and low (Fig. 2D) kidney methylation variability across individuals. Among all cases, uniquely methylated sites demonstrated the strongest spatial separation. We concluded that uniquely methylated sites can be utilized for unbiased characterization of kidney functional decline.

### Multiple liver diseases show loss of epigenetic information

The liver is renowned for its exceptional regeneration capacity, which may make it less susceptible to aging effects compared to other tissues. Indeed, the risk of some liver diseases does not appear to correlate with age, at least in men [26, 27]. However, liver function declines with age, and aging is associated with increased severity and poor prognosis of multiple liver diseases [28]. To explore the role of epigenetic information loss in liver diseases, we analyzed a combined multi-liver-disease and multi-tissue dataset ([29]; GSE61256; *N* = 137; Supp. Tables 2–3). It includes, in addition to liver methylation samples from healthy individuals, data for healthy but obese participants, as well as Primary Biliary Cirrhosis (PBC), Non-Alcoholic SteatoHepatitis (NASH), Primary Sclerosing Cholangitis (PSC) and Non-Alcoholic Fatty Liver Disease (NAFLD) patients. Methylation data for adipose and muscle tissues were also available for some donors. Our tissue-specific dataset contains 3,673 liver-specific methylation sites, all of which were found in this dataset, enabling high-confidence detection of epigenetic information loss. To exclude the possibility of technical variations affecting the analysis, we measured the correlation between liver methylation in the NGDC-CNCB dataset, used to derive the unique sites, and the controls of GSE61256, and found it to be *r* = 0.99 (Supp. Figure 1 A), indicating high technical reproducibility across different datasets. We first explored whether methylation levels in these sites correlate with all liver diseases. Remarkably, liver-unique methylation sites exhibited a bimodal distribution with a positive (predominantly blue dots) and a negative peak (predominantly red dots), and almost no sites showed low correlation to liver disease (Fig. 2E, discontinuous presence on the Y-axis). In contrast, the same sites in the same patients, but in adipose tissue, showed a normal distribution (Fig. 2E, continuous presence on the X-axis). We investigated what distinguishes sites that positively vs. negatively correlate with liver disease. Overwhelmingly, these two populations were distinguished by their methylation status in the liver compared to the rest of the body (3,658 of 3,673; *p* < 10^–1000^; blue vs. red in the plots). Sites that displayed lower methylation in the liver, as compared to the rest of the tissues (blue), had higher methylation values in diseased livers (positive correlation), while sites with higher liver methylation (red) had decreased methylation in diseased livers (negative correlation). Thus, 99.6% of these sites regressed to the mean with liver disease progression (Fig. 2F–G).

Contrary to our expectations based on these results, PCA of these samples showed a poor separation between healthy and diseased livers (Supp. Figure 1B). We hypothesized that another source of variance was dominating this analysis. Indeed, we found that men and women formed two distinct clusters when performing PCA using high-variability sites (Supp. Figure 1 C), in line with previous findings that the human liver has a sex-specific methylation profile in autosomes [30]. When analyzing men separately, controls clustered together but did not completely separate from diseases (Fig. 2H). Control methylation sites exhibited weaker separation of the control group (Supp. Figure 1D). Surprisingly, analyzing women only yielded a relatively poor separation of controls (Supp. Figure 1E). That can be attributed to gene expression differences between men and women [31–33]. These inherent dissimilarities might manifest in methylation profiles and different hormone levels in the liver [34], and the nature of PCA analysis which segregates samples according to maximum variance and not by a certain biological orientation. These results suggest that liver disease results in epigenetic information loss in the liver.

### Type 2 diabetes shows epigenetic information loss in adipose but not in the pancreas

**Fig. 3:**
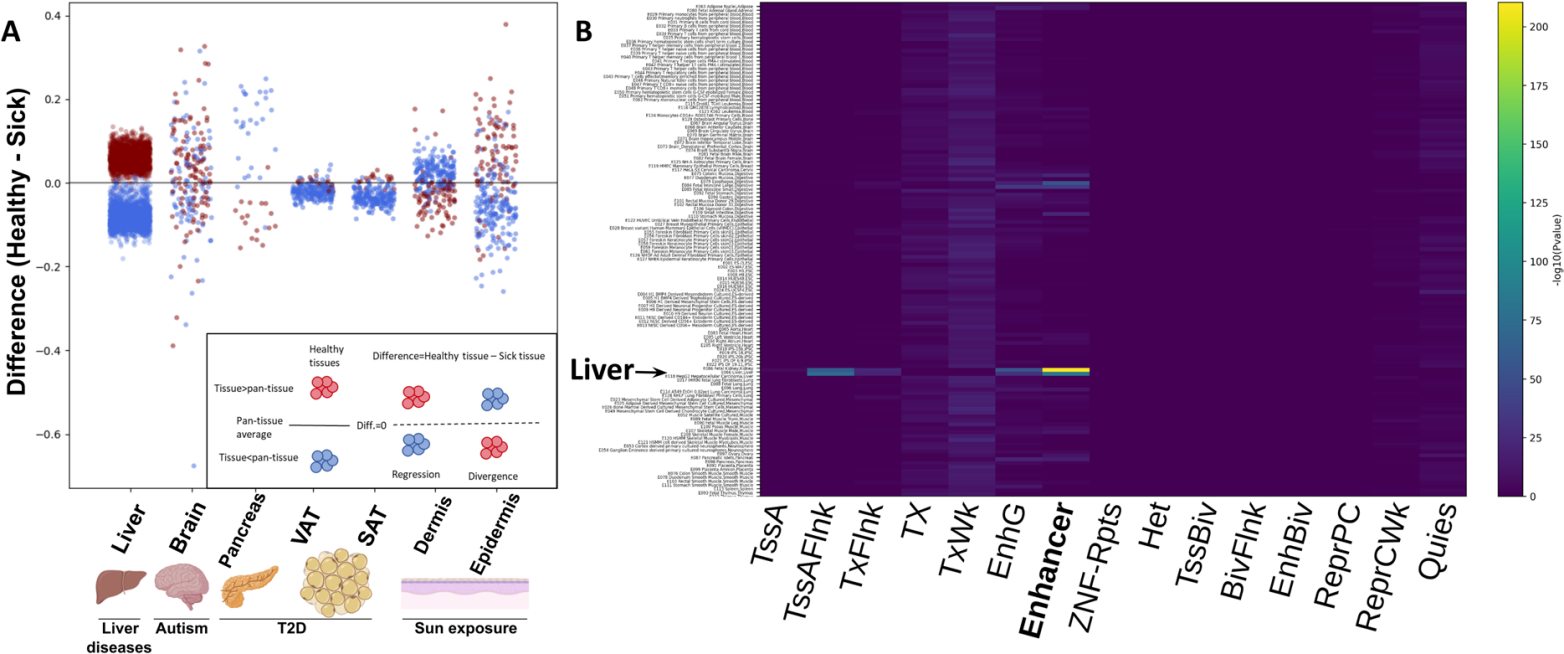
Methylation difference in disease across seven tissues. (**A**) Inset—schematic explanation: Red dots represent sites methylated above other tissues, while blue dots represent lower methylation levels. When looking at the difference between healthy and sick, red above zero and blue below indicate regression to the mean, while the opposite indicates divergence. Y-axis: Difference in methylation levels, for each tissue-unique methylation site, between healthy and sick (cohort averages). Differences shown for liver (liver diseases), Brain (autism), pancreas (T2D), VAT and SAT (T2D) and dermis and epidermis (sun exposure). (**B**) Enrichment of liver unique (low in liver) methylation sites across 15 chromatin states (x-axis) and 127 tissues and cell lines (y-axis). Strongest enrichment, highlighted with “liver” text and arrow, was observed for methylation in liver tissue (E066), followed by hepatocellular carcinoma (E118)

T2D is the most prevalent form of diabetes, characterized by high blood sugar, insulin resistance, a relative lack of insulin, and changes to the pancreas [35–37]. Age is a major risk factor for T2D, and it correlates with increased biological age as indicated by epigenetic clocks [38]. To investigate if epigenetic information is lost in the pancreas of T2D patients, we applied our analysis to a dataset mapping 27,578 CpG sites in human pancreas samples from T2D patients and controls ([39]; GSE21232; *N* = 16). Of the 705 pancreas-unique methylation sites, only 45 were covered by the methylation array used. Among these, 28 were less methylated in the pancreas. Contrary to our findings in liver and kidney, 26 of the 28 sites diverged away from the mean in T2D (Fig. 3A; *p* < 10^–5^). Of the 17 sites that were more methylated in the pancreas, divergence was observed in 16 (Fig. 3A; *p* < 10^–3^). PCA almost completely separated diabetic from non-diabetic pancreas samples (Supp. Figure 2 A). Interestingly, using random sites or sites with high variance in the pancreas—but not sites of low variance—also separated diabetic from non-diabetic pancreases (Supp. Figure 2B). Thus, the epigenetic changes are robust enough for T2D to be detected in multiple sites.

Diabetes is closely linked to obesity and the accumulation of adipose tissue. However, not all adipose tissues confer the same risk. Specifically, visceral adipose (VAT) poses a significantly greater risk than subcutaneous fat (SAT) [40]. To test if epigenetic information is lost in diabetes, we examined a dataset of VAT and SAT from T2D patients and controls ([41]; GSE162166; *N* = 40). Globally, some methylation patterns changes were observed. In VAT, 84% of all methylation sites showed elevated methylation in T2D patients. Furthermore, examining methylation sites with a robust change in any direction—measured by an absolute correlation (methylation vs. disease status) greater than 0.3—94% of sites showed increased methylation. Interestingly, this was observed in VAT only, whereas in SAT, only 46% showed elevated methylation. Adipose tissue has 222 unique sites (Fig. 1D, Supp. Tables 2–3). Among these, 212 were covered by the methylation profiling dataset, including VAT and SAT from T2D patients and controls ([41]; GSE162166). Methylation patterns regressed towards the mean at 90% of these sites in VAT and 95% in SAT (Fig. 3A—blue dots below the zero line and red dots above it). Although statistically significant, this finding is not particularly surprising, as 91% of the unique sites exhibited lower methylation levels in adipose tissue, implying that the general trend in VAT can largely account for this observation. Considering the remaining 9% (*N* = 18) of the sites, regression towards the mean (and contrary to the 84% trend observed) was evident in 9 of them (*p* = 10^–3^). In SAT, 14 of the 18 sites regressed towards the mean (*p* < 10^–7^). Consequently, we concluded that a global increase in methylation is a characteristic feature of VAT, but not SAT, in T2D patients, and both tissues experience a loss of epigenetic information. However, these findings were not accompanied by clear separation by PCA, as healthy and T2D patients did not form distinct clusters in neither VAT nor SAT, when using adipose-unique sites (Supp. Figure 2 C).

### Autism does not show significant epigenetic information loss

Autistic Spectrum Disorders (ADS) is a neurodevelopmental disorder characterized by difficulties with social interaction and communication. ASD has high concordance among monozygotic twins (> 90%) compared to dizygotic (~ 50%), indicating a strong genetic component and complex inheritance. [The continuing value of twin studies in the omics era. Nat Rev Genet, 2012. 13(9): p. 640-53]. Symptoms of autism typically appear early in childhood, and it has not been linked to aging. Thus, serving as a negative control to our aging-associated information loss hypothesis, we tested if epigenetic information is lost in autism. The brain contains a relatively large number of unique methylation sites: 6,491. Of these, 209 were covered by a dataset of brain methylation ([42]; GSE38608; *N* = 36). In autism, we observed information loss in 125 of these. While these findings were marginally statistically significant (*p* = 0.005), they appeared very weak compared to kidney and liver diseases (Fig. 3A—no clear separation of red and blue above or below the zero line in brain, unlike in the liver). We performed PCA using all brain-unique methylation sites, and autistic brains appeared indistinguishable from controls (Supp. Figure 2D). A clear signature was observed, but it was not related to autism (see “Epigenetic information loss during aging” below). Together, these results suggest that loss of tissue-specific methylation information is not a universal feature of all diseases and disorders. However, these results do not exclude the possibility that a different analysis or sample correction may reveal information loss.

### Epigenetic information is lost in environmentally stressed tissue

The skin serves as a barrier that protects the body from external insults, such as UV radiation from the sun. Sun exposure prematurely ages the skin, termed photoaging [43]. Photoaging manifests differently in the dermis and epidermis, which can be partly attributed to the limited penetration of UV-B into the dermis [44]. We investigated whether typical environmental stressors to tissue induce epigenetic information loss. To do so, we compared methylation data from sun-exposed and sun-protected skin samples of both dermis and epidermis, in young and old individuals [45]. Of the 277 skin unique methylation sites, 275 were present on the dataset (GSE51954; *N* = 78). In the dermis, divergence from the mean upon sun exposure was observed (Fig. 3A—dermis. Blue dots above zero, red below; Supp. Table 3). In contrast, in the epidermis, regression to the mean occurred at 223 and 226 sites for young and old individuals, respectively (Fig. 3A; Supp. Table 3). When mapping methylation-levels of skin-unique methylation sites into 3D space using PCA, dermis and epidermis samples were clearly separated (Fig. 2I). However, samples from older versus younger donors exhibited only partial separation (Fig. 2I). Sun-exposed and sun-protected samples from each tissue type clustered separately, with the expected stronger separation observed in the epidermis. Analyzing the same samples using methylation sites that were not tissue-specific, including random sites and sites with high standard deviation, did not yield such a distinct separation (Fig. 2J–L). Consequently, we conclude that sun exposure is associated with epigenetic information loss in the epidermis.

### Epigenetic information loss during aging

**Fig. 4:**
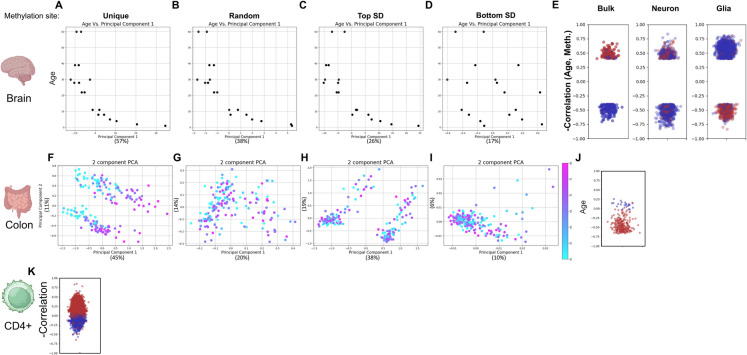
Epigenetic information loss in aging. PC1 vs age in brain samples, using (**A**) unique (**B**) random (**C**) high variability and (**D**) low variability sites. (**E**) Correlation of methylation levels and age for brain-unique methylation site, in bulk, neuronal and glial cells. PCA of intestine samples, using (**F**) unique (**G**) random (**H**) high variability and (**I**) low variability sites. Color indicates age. (**J**) Correlation of methylation levels and age for intestine-unique methylation site (**K**) Correlation of methylation levels and age for intestine-unique methylation site. All correlations are plotted inversely for schematic consistency (red above zero for regression). In each plot, the number of sites in the control groups was selected to match the unique group

The epigenetic theory of aging posits that the loss of epigenetic information contributes to the aging process [10, 13, 46, 47]. Tissue functionality declines with age, similar to the deterioration observed in multiple diseases. This decline may vary among tissues, as some experience a faster loss of function on average than others. Nonetheless, a strong epigenetic signature of aging has been observed in most tissues [5]. We tested if tissue-unique epigenetic information is lost during aging, focusing on three tissues. The brain has low regenerative capacity and slow cell turnover. With aging, cognitive functions decline and memory formation may become impaired. In contrast, the intestine exhibits high regenerative capacity and cell turnover. Aging in the intestine is characterized by reduced self-renewal capacity of intestinal stem cells and diminished tissue self-repair functionality [48]. When plotting the brain-unique methylation sites for autistic and nonautistic individuals, we noticed PCA separated the samples by age (Supp. Figure 2E and Fig. 4 A). Indeed, PC1 strongly correlated with the age of the individuals (Fig. 4A). However, random sites, as well as sites with high variance also produced the same result (Fig. 4B–D). Of the 209 brain-unique sites covered by the dataset, regression to the mean was observed in 148, and not observed in 61 sites (*p* < 10^–9^). Next, we analyzed normal colon mucosa samples ([49]; GSE48988; *N* = 178). Of the 3894 intestine-unique sites, 290 were found in the dataset. Of these, 87% (252) had higher methylation levels in the intestine than in other tissues. 48 sites did not produce any methylation values and were discarded. Divergence from the mean was observed in 237 of the 242 remaining sites, while regression was observed in just 5 sites (*p* < 10^–63^; Fig. 4J). PCA generated a clear age gradient when using intestine-unique sites, but not in any of the controls (Fig. 4F–I). We concluded that the tissue-unique epigenetic signature degrades with age, but only in some tissues. This result is independent of whether unbiased PCA clustering separates samples by age.

### Functional analysis of tissue-unique methylation sites

Assigning functional significance to the methylation sites underlying epigenetic clocks remains challenging [50]. We have previously shown that kidney-unique methylation sites are enriched for disease relevant transcription factors and the less-methylated sites are enriched for kidney tissue-specific enhancers [23]. To test if this is a general attribute of tissue-unique methylation sites, we selected from the 30 tissues analyzed for unique methylation sites (Fig. 1D; Supp. Table 1) and identified the tissues most similar to the seven disease tissues investigated (Fig. 3A). The low-in-tissue (blue) methylation sites were analyzed using eFORGE 2 for enrichment of 15 chromatin marks across and multiple samples [51]. In all tissues analyzed, the strongest enrichment was observed for tissue-specific enhancers of tissues most proximal to the analyzed tissue (Fig. 3B, Supp. Figure 3 A) [52]. For example, sites showing low methylation in liver and high in all other tissues were most enriched for enhancers identified in liver tissue (Fig. 3B). The only exception to this was in brain tissue, where strong enhancer enrichment was additionally observed in ES-derived ectoderm CD56 + cell culture, the primary germ layer of origin for most brain cells, with a marker crucial for neuronal development, as well as ES-derived neuronal progenitor cells (Supp. Figure 3 A). We concluded that unique undermethylated sites are enriched for tissue-specific enhancers, and these get more methylated in some age-associated diseases. Similar findings were reported for cell-specific unmethylated regions [25]. Next, we tested for enrichment of known transcription factors in each tissue. Tissue-unique methylation sites showed enrichment for known transcription-factor binding sites. Interestingly, different tissues showed enrichment for different transcription factors, with a limited overlap between motifs identified in different tissues (Supp. Figure 3B).

### Epigenetic information is lost in some, but not all, cell types

To investigate whether epigenetic information loss occurs within a cell population, we analyzed a dataset of human brain samples ([53]; GSE41826; *N* = 145) which included bulk tissue samples as well as fluorescence-activated cell sorted (FACS) neuronal and non-neuronal (predominantly glia) cells. In the bulk samples, consistent with our previous observation, regression to the mean with age was observed in 85% (1302/1531; *p* < 10^–181^) of more methylated sites and 77% (3819/4926; *p* < 10^–344^) of less methylated sites. Analyzing only sites that robustly correlate with age (*R* > 0.4) increased these numbers to 96% (*p* < 10^–31^; Fig. 4E) and 86% (*p* < 10^–30^) respectively. This trend was mimicked in neurons, where regression to the mean was observed in 71% (*p* < 10^–9^) and 70% (*p* < 10^–32^), of more and less methylated sites, respectively. Surprisingly, the opposite trend was observed in glia cells, where methylation levels predominantly diverged away from the mean (85%; *p* < 10^–49^ and 94%; *p* < 10^–390^, respectively).

To further corroborate these findings, we analyzed the NGDC-CNCB blood DNA methylation dataset [54]. This independent dataset comprises 25 types of blood samples (*N* = 3402), including mixed and cell-type-specific samples. We focused on purified naive CD4 + T cells (CD4 +). As these cells were not present in our original analysis, we identified 29,137 methylation sites unique to CD4 + cells (Supp. Table 4), compared to the remaining 24 blood sample types. Correlation of methylation level and age was calculated for each unique site for samples where age was available (*N* = 71). The majority of the sites were more methylated in CD4 + compared to the remaining samples. Of these, 96% (20825/21556; *p* < 10^–1000^) of sites regressed towards the mean with age (Fig. 4K—red dots). Similarly, of the less methylated sites 93% (7102/7581; *p* < 10^–1000^) regressed towards the mean (Fig. 4K—blue dots). Thus, some cell types regress to the mean, while others diverge away from it.

### Methylation-based disease classification

Tissue-specific methylation signature changes in various diseases raise the question of whether these unique sites can be used for disease classification. We applied three classification models using only methylation data. These were projected to 2D space using PCA. First, we looked at the ratio of average distances from the analyzed sample to healthy and sick samples. Second, we compared which is larger, the distance from the analyzed sample to the control group center of mass or the disease group center of mass. Third, if the distance from a sample in 2D space was less than twice the standard deviation from the mean location of the disease group, the sample was classified as sick. We were able to distinguish between low and high GFR (kidney) with an accuracy as high as 82.35%, between sun-exposed and sun-protected skin samples in the dermis with an accuracy of 80%, and in the epidermis with an accuracy of 100%, respectively (Supp. Figure 4).

## Discussion

Aging is accompanied by epigenetic changes. By focusing on DNA methylation sites with unique signatures compared to other tissues, we could isolate genomic loci where age-dependent changes in methylation can be interpreted. In these sites, observing the direction of change can inform us if it is random, hypomethylation, hypermethylation, divergence or regression to the mean. In multiple instances, including diseases, environmental stress, and natural aging, these sites predominantly changed by regression to the mean. For example, 99.6% of liver-unique sites regressed to the mean in liver diseases. We interpret these results as a loss of epigenetic information. The unique methylation signature that characterizes the tissue becomes more similar to other tissues in the body, akin to the smoothening of the epigenetic landscape or exdifferentiation reported elsewhere [11, 13, 55]. As age is a risk factor for multiple diseases, we needed to exclude the possibility of misattributing age-related changes to diseases. Indeed, in the analyzed disease cohorts, disease, and not age, was the factor able to explain the observed changes. These results do not imply that a similar process does not occur in other methylation sites. On the contrary, the fact that such a large fraction of the a-priori selected sites showed this trend suggests this is likely to be a more general phenomenon. Moreover, other types of epigenetic information may exhibit a similar phenomenon. However, analyzing these sites will require the development of expectations as to the direction of change, to distinguish information loss from changes that may reflect different processes. Interestingly, some tissues that do not show this phenomenon demonstrate the opposite effect. Methylation sites diverge away from the common form as the tissue becomes more distinct. Thus, our experiments do not support a role of epigenetic information loss, at least for these sites and bulk tissue. We postulate that another unknown mechanism, actively counteracting the smoothening of the epigenetic landscape, is dominant in these samples.

We found that tissue unique methylation sites are enriched for enhancers specific to the same tissue (Fig. 3B and Supp. Figure 3 A). These sites tend to become more methylated, and thus typically less accessible, with the progression of some age-associated diseases (Fig. 3A). One could speculate that this closure of tissue-specific enhancers leads to loss of tissue-specific chromatin architecture and drives the smoothening of the epigenetic landscape. However, additional data is needed to infer causality and substantiate such a speculation.

One possible explanation for the loss of epigenetic information in disease and aging is changes to tissue cell composition. Such changes, including replacement of resident-tissue cells by fibroblasts and accumulation of immune cells, have been demonstrated in aging and disease in some tissues, but not in others [56–59]. A non-exclusive alternative is changes within the cell population. For example, in epithelial to mesenchymal transition (EMT), epithelial cells undergo changes that enable them to assume a mesenchymal cell phenotype [60]. EMT has been associated with fibrosis in kidney, liver, and intestine [60–64] and changes in DNA methylation have been suggested to causally underlie EMT [65]. In human brain samples, regression to the mean with age was observed in brain-unique sites of bulk tissue. When looking at sorted cells, we observed regression to the mean in neurons, but not glial cells of aging brains. In glial cells methylation levels diverged away from the mean, and the tissue-unique signature appears to become stronger. Similarly to neurons, regression to the mean with age was observed in unique sites in CD4 + cells. Future analysis of samples separated by cell type will enable us to determine the relative contribution of each of these mechanisms to epigenetic information loss, as these may differ across organs and pathologies.

Epigenetic information holds diagnostic value. Using the same algorithms and solely relying on methylation data, we were able to classify diseased and healthy organs at high rates. These results highlight the diagnostic potential of methylation data. As some disease induced changes to the methylome are reversible [7], and heterochronic parabiosis can reverse or delay some changes to epigenetic age [8], it would be interesting to investigate if treatment may reverse the regression to the mean in affected organs. Moreover, it has the potential to suggest which organs are affected by a medical condition. More sophisticated analyses may even distinguish different diseases in the same organ and help classify new disease subtypes. Finally, the epigenetic signature of these tissue-specific sites may allow for their identification from cell-free DNA in blood.

Epigenetic drift, characterized by increased variability and stochastic changes in DNA methylation patterns with age and disease, has been well-established in the literature [66]. Recent studies have shown that such stochastic changes alone are sufficient for generating aging clocks [67]. However, it has also been demonstrated that non-stochastic processes drive a large portion of the changes observed in some clocks [68]. Our results strongly support the latter finding. By pre-selecting and focusing on tissue-specific methylation sites, we reveal a directional change in methylation patterns, rather than the random variations associated with epigenetic drift. Specifically, we demonstrate that these preselected sites overwhelmingly change in a predicted manner, with tissue-unique methylation patterns regressing towards a mean state in multiple diseases and some aging tissues. This holds true even when we exclude sites that trend towards any global average (Fig. 2F). Our methodological approach allows us to interpret these changes as a loss of epigenetic information, offering a new framework for understanding the functional implications of age- and disease-related methylation changes.

Major limitations of this work include predominantly bulk sample analysis, with limited cell-type and no single-cell data, and lack of longitudinal data. We focused on tissue-level methylation levels across multiple sites. However, each tissue is composed of multiple cell types, often arranged in functionally-important spatial patterns. The bulk analysis limits the extractable information. Cell-type unique methylation sites may have remained undiscovered in our analysis as the signal was suppressed by other cells of that tissue. Similarly, changes that manifest only in a subset of cells may not be detected. For example, beta cells are lost in T2D. However, as they comprise only 1–2% of the pancreas mass, their loss in T2D is unlikely to be captured by this analysis. Indeed, our analysis of pancreas-unique sites identified significant enrichment for pancreas enhancers; however, no enrichment for pancreatic islet enhancers was observed (Supp. Figure 3 A). Thus, these experiments do not inform us if epigenetic information loss plays a role in beta cell loss in T2D. The addition of such cell-specific information, both for unique methylation site identification and for deeper tissue analysis, will enable greater sensitivity while informing us which cells are losing information and by which mechanism. Another limitation is array design, which particularly for the 27 K array, potentially skews the results towards hypomethylated probes and does not provide a balanced representation of the changes in the epigenetic landscape. Next, while we were able to demonstrate that methylation-based disease classification is possible in some cases, our study focused on comparing disease samples to control samples. Future studies should aim to identify changes in methylation patterns within individuals over time, as this would enable us to identify epigenetic information loss in an individual rather than in comparison to a control. Other algorithms are also likely to produce better results, as we did not attempt to optimize for disease detection, but merely to show that such information loss correlates with disease state. Finally, we note that while aggregation of hundreds to thousands of sites across tens of samples enabled robust statistics, the average data content per individual site and patient is low. This is likely the reflection of the stochastic nature of this process.

## Methods

### Unique methylation sites

A site was considered unique for a tissue if the average methylation value in that tissue was either (i) at least 0.1 higher than the 95% quantile of all other tissues (combined) or (ii) at least 0.1 lower than the 5% quantile of all other tissues in the NGDC-CNCB dataset. CD4 + unique sites were identified by applying the same analysis to the NGDC-CNCB blood dataset. Methylation sites with fewer than 2,000 valid values in the dataset were omitted from the analysis. The dataset, containing Illumina HumanMethylation 450 BeadChip probes, was screened for the above criteria using a custom Python script. The results were manually verified for a random subset. Among the 30 available tissues, 8 did not have unique sites. It should be noted that these criteria do not entirely eliminate the possibility of two tissues sharing the same unique site. Parsing the dataset, analyzing the data and sorting the unique sites was done using Python.

The resulting dataset was checked against lists of cross-reactive probes [69, 70]. Less than 4% of the unique methylation sites identified were present in this list. Of these, 80% were placenta sites. In analyzed tissues, tissue-unique CpGs contained less than 3% cross-reactive probes. Of these 3%, almost none were in the high number of “bases matched to cross-reactive targets” group. Removing these probes did not significantly change any p-value. We also compared tissue-unique probes to hypervariable probes [71]. We found that the ICC scores of liver unique methylation sites follow the exact same distribution as the rest of the array (Supp Fig. 5). Limiting the liver analysis to probes with higher ICC scores reduced the number of probes and thus the resulting *p*-values, but did not affect the overall result. Finally, we observed no statistically significant overlap between colon, breast, and kidney unique probes and the corresponding tissue-specific mQTLs [69].

### Datasets used

Methylation data were obtained from the Gene Expression Omnibus [1, 72]. All datasets used are listed in Supplementary Table 2. Analysis was conducted on the processed Series Matrix Files, which were separated into a methylation data file and an experiment metadata file.

### Motif enrichment

We analyzed the unique sites for de novo and known motifs across all tissues using Hypergeometric Optimization of Motif EnRichment (HOMER) [73, 74]. We extracted ± 500 bp around the start of methylation probes using the Python Bio.Entrez package [75], and obtained chromosome, strands, and genomic coordinate data from the HM450 hg19 database. Sequences were obtained for all unique methylation sites across all tissues and for 3,000 random methylation sites, which were used as background for motif enrichment detection. Sequences were saved in Fasta format using Python. HOMER was run for each tissue’s unique sites separately, against the background sequences, and the motif results were filtered by *p*-value < 10^–3^ and occurrence percentage in the background < 10% using a Python script. Enrichment was calculated as the log2 of the percentage in the target divided by the percentage in the background. These data were used to generate Supp. Figure 3B.

### Chromatin state classification

We analyzed the unique low-in-tissue (blue) using eFORGE 2 (https://eforge.altiusinstitute.org/; [51]) with the following setting: “–format probeid –bkgd 450 k –data erc2-chromatin15state-all –noproxy –reps 1000 –thresh 0.05,0.01”. In cases where the number of unique sites exceeds the capacity of the software (brain), 1,000 sites were randomly selected and used for the analysis.

### Principal component analysis

PCA linear dimensionality reduction was employed to project n-dimensional data (n CpG sites) onto 2D/3D space. We utilized the Python scikit-learn package to perform PCA. The data were parsed and rearranged from a dataframe format to comply with the package's format requirements, representing probes as dimensions. Whenever a subset of the database was required, we either reconstructed the input files to include only the required dataset or filtered out test subjects in the code prior to implementing PCA on the data. Filtering was conducted on metadata stored in a separate dataframe and/or on methylation sites.

### Disease classification

Values for unique methylation sites were extracted from the databases for each relevant tissue analyzed in the respective datasets and projected onto 2D using PCA. Samples were classified using three models: 1) Ratio of average distances from the sample to healthy and sick samples. 2) Comparing distance from the sample to the control group center of mass and distance from the sample to the disease group center of mass. 3) If the distance from a sample in 2D space was less than twice the standard deviation from the mean location of the disease group, the sample was classified as sick.

To estimate the accuracy of each model, in each iteration, one sample was omitted from the PCA analysis and then transformed and projected onto 2D space using the PCA weights vector. The three models were then applied to each sample, and accuracy was calculated by comparing classification with true results from the database.

All the models projected a single sample onto an existing PCA space based on the rest of the samples, and subsequently classified it into one of the groups without prior knowledge.

### Statistics

*P*-values were calculated using the binomial distribution in R (dbinom), with the null hypothesis that the direction of change is random (probability = 0.5), except when otherwise specified. We note that as we analyze less and more methylated sites independently, introducing any other value, for example to account to global demethylation, would lower the p-values of one group but will increase the p-values of the other. Where applicable, technical duplicates were removed from data before the analysis.

## Supplementary Information

Below is the link to the electronic supplementary material.

Supplementary file1 (DOCX 2333 KB)

Supplementary file2 (XLSX 4022 KB)

Supplementary file3 (XLSX 6 KB)

Supplementary file4 (XLSX 17044 KB)

Supplementary file5 (CSV 1815 KB)

## Data Availability

Generated data are available as supplementary files.
