# Supplementary material for: Epigenetic information loss is a common feature of multiple diseases and aging
Source: GeroScience. 2025 Jul 11;48(2):2311–25. doi: 10.1007/s11357-025-01767-7 (PMC12972351; doi:10.1007/s11357-025-01767-7)
Supplement: Supplementary file 1 — Supplementary file1 (DOCX 2333 KB) [file 11357_2025_1767_MOESM1_ESM.docx]

**Supplementary figures**

**
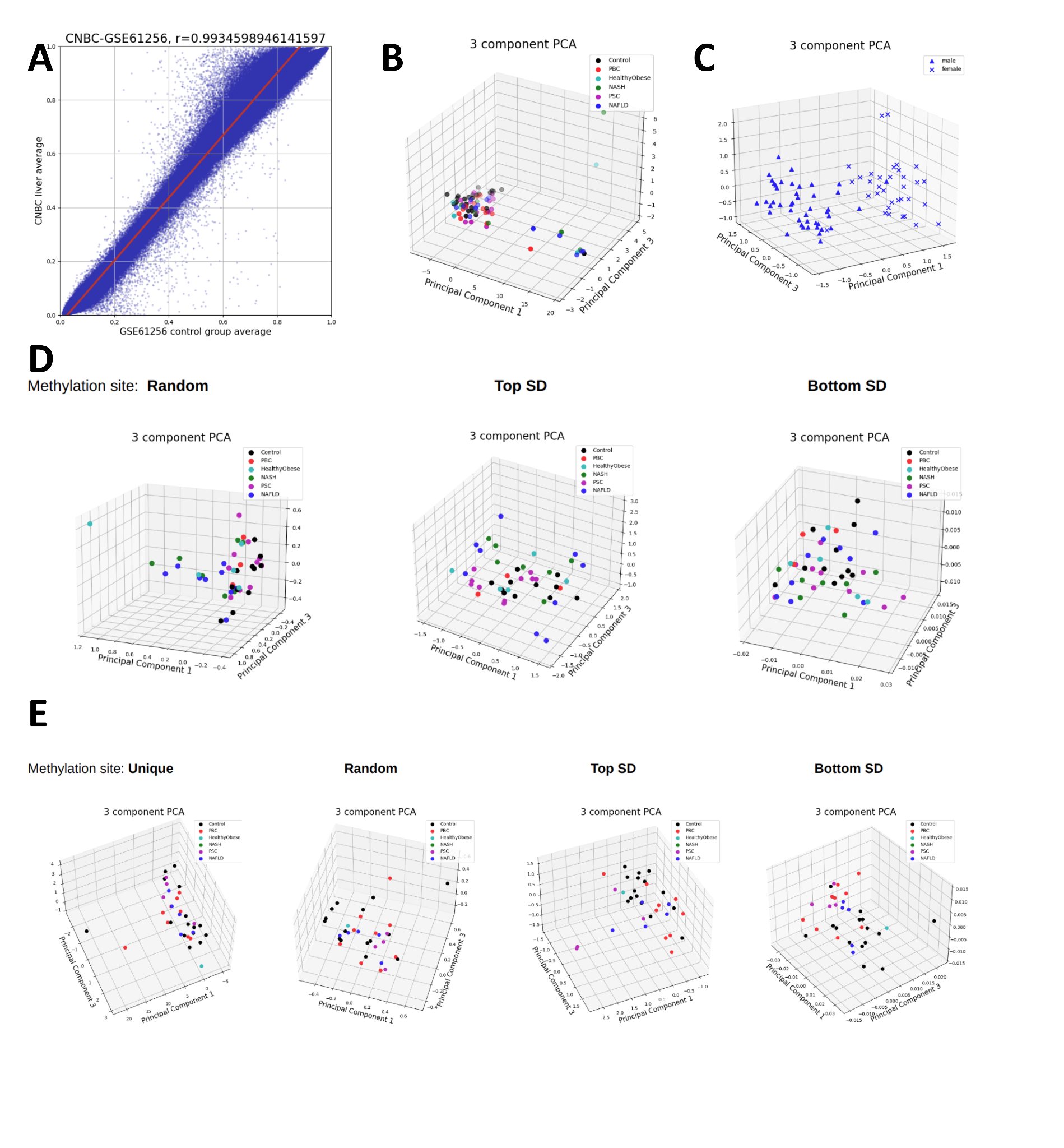
**

**Supplementary Figure 1. (A)** Pearson correlation of average liver methylation values in the GSE61256 and the NGDC-CNCB datasets. **(B)** PCA analysis of all GSE61256 liver samples, using uniquely methylated sites. **(C)** PCA analysis of all GSE61256 liver samples using high-variability sites. **(D)** PCA analysis of male liver samples from GSE61256, using random, low-variability and high-variability methylated sites.(**E)** PCA analysis of female liver samples from GSE61256, using unique, random, low-variability and high-variability methylated sites. In each plot, the number of sites in the control groups was selected to match the unique group.


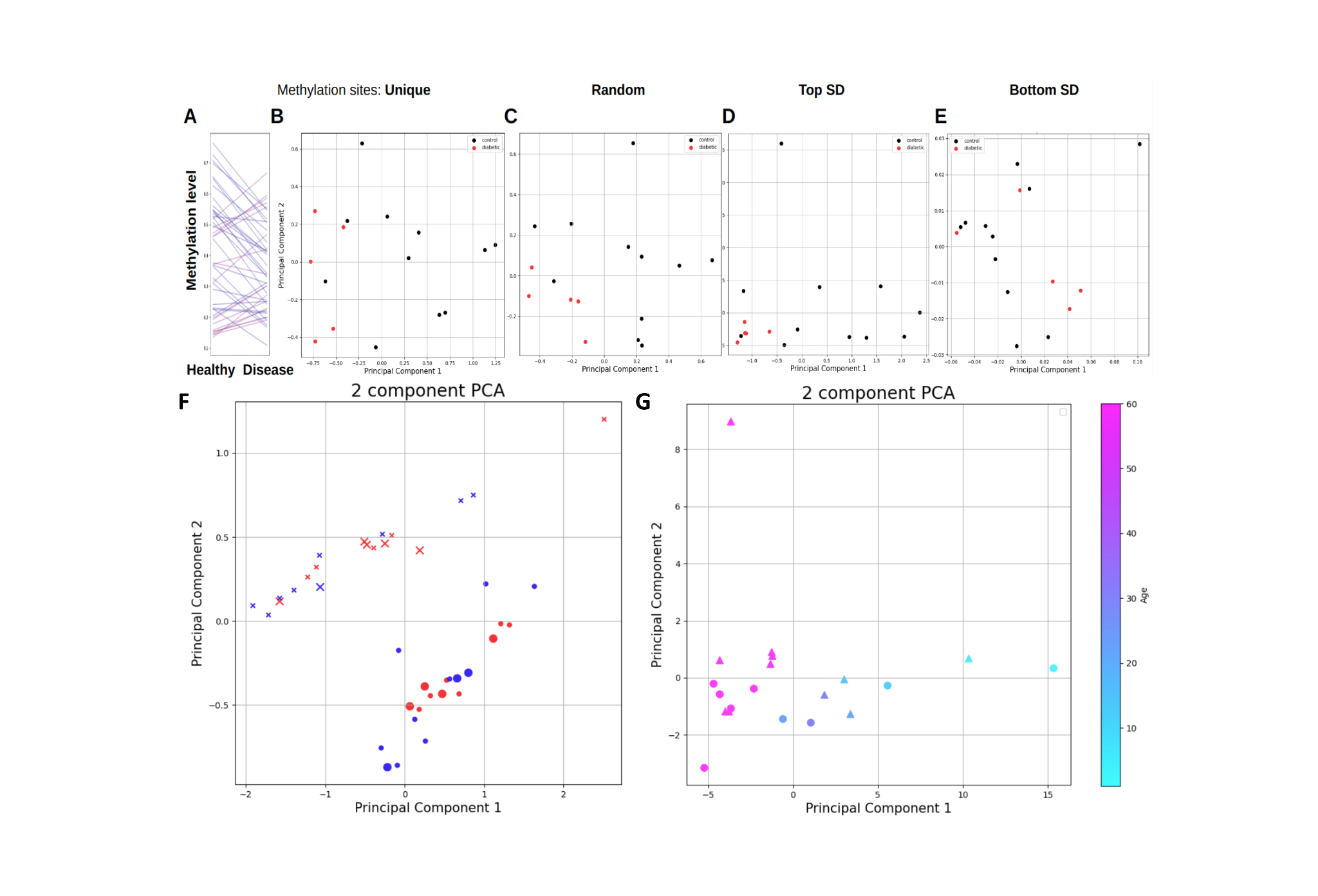


**Supplementary Figure 2A. Tissue-unique sites in the pancreas of T2D patients.** (**A**) Pancreas-unique methylation sites diverge from the mean both for sites that are more (red) and less (blue) methylated. PCA analysis separates healthy (black) from T2D pancreas when using pancreas-unique (**B**) random (**C**) and high variability (**D**) sites, but not low variability sites (**E**).**(F)** PCA mapping using tissue-unique sites in the adipose of T2D patients. Healthy - blue; diabetic - red. SAT - x; VAT - circle. Size is sex: small - female, large - male.**(G)** PCA analysis of brain samples from GSE38608, using uniquely methylated sites. Circles represent control samples, while triangles represent autism samples. Colors indicate age. In each plot, the number of sites in the control groups was selected to match the unique group.


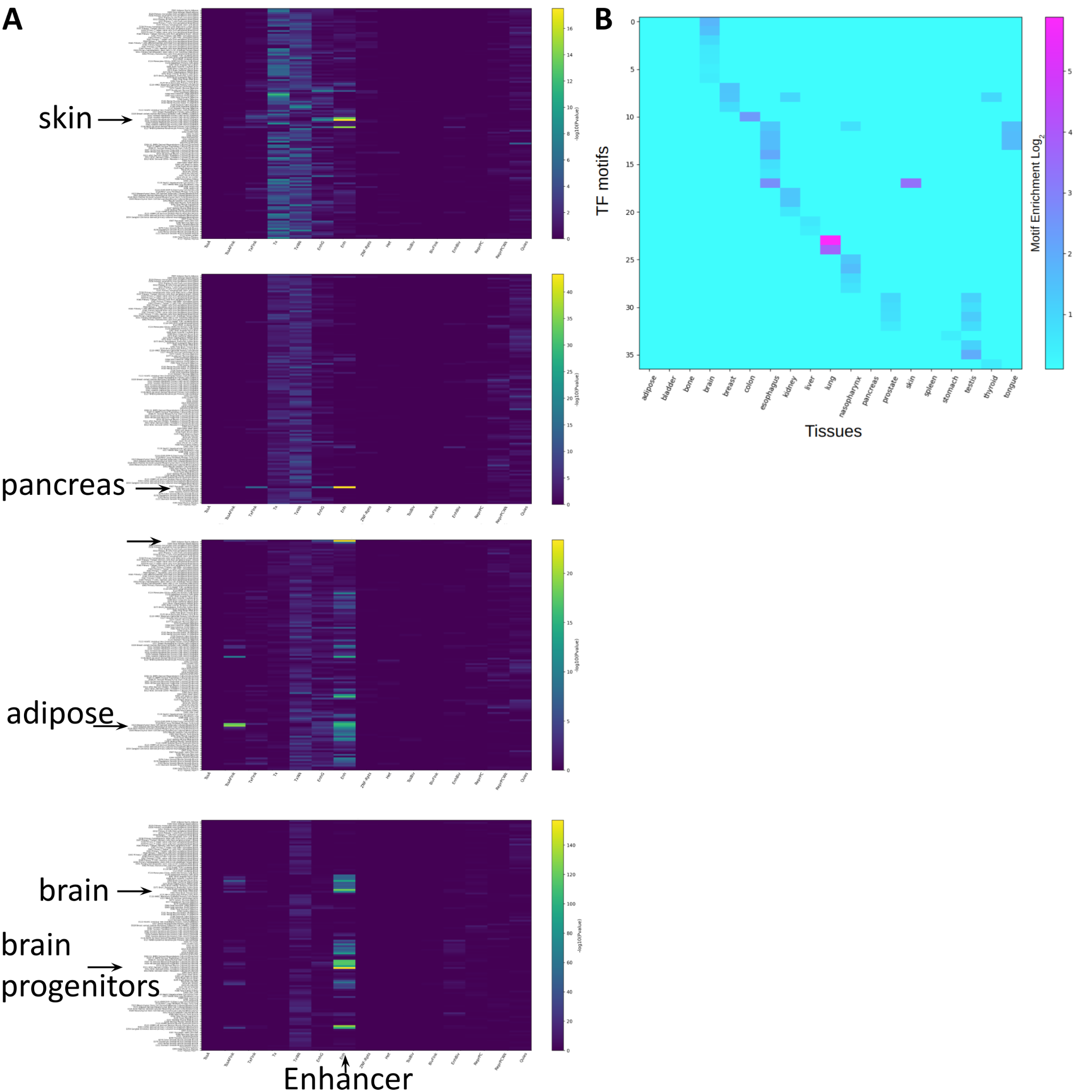


**Supplementary Figure 3.** (**A**) Enrichment of unique-low methylation sites across 15 chromatin states (x-axis) and 127 tissues and cell lines (y-axis). Skin, pancreas, adipose and brain are shown in descending order. Arrows and text mark tissues with high enrichment, these match the tissues from which the unique sites were derived from. Enhancer chromatin state column is also text labeled. Full details of chromatin annotation marks can be found at [^52^](https://paperpile.com/c/WBmJhM/oiZen) (**B**) TF motif enrichment in different tissues.

**
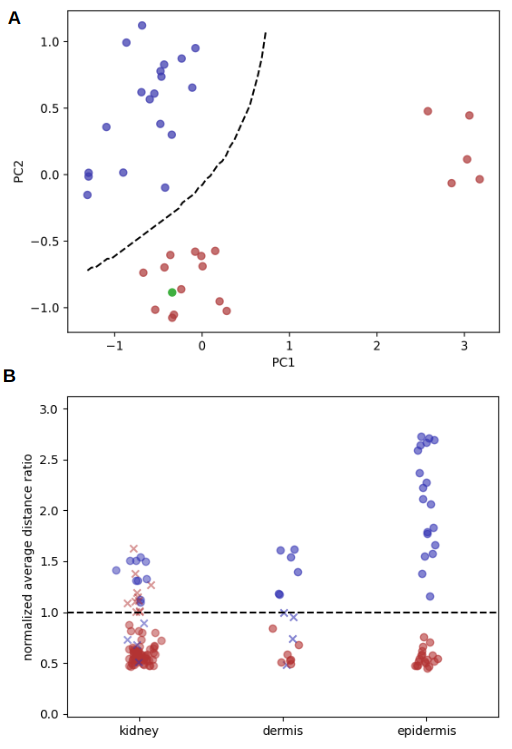
**

**Supplementary Figure 4. (A)** Classification of a single epidermis sample (green dot) in leave-one-out analysis using 2D PCA. The separating (dashed) line was calculated using the formula
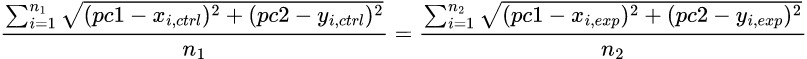


**(B)** Kidney filtration rate and skin exposure classification using PCA and average distance ratio in leave-one-out cross validation analysis. Red dots show high eGFR/ sun exposed skin, while blue dots show low eGFR/ sun protected skin. Correct decisions are marked by full circles whereas incorrect decisions are marked by x.


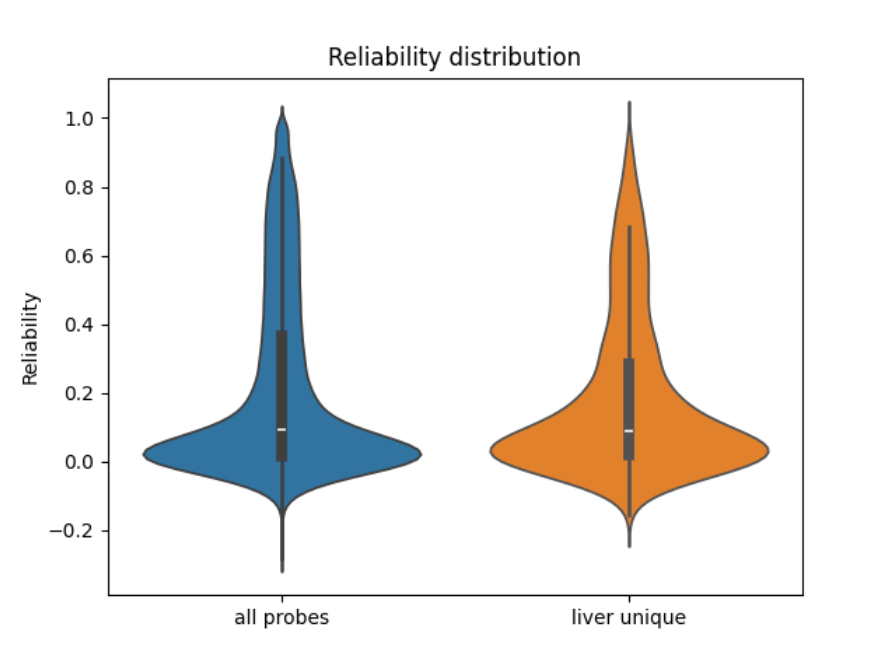
**Supplementary Figure 5.** ICC score distribution of all array probes and liver-unique probes.
